# CRISPR/Cas9-mediated knockout of Mct8 reveals a functional involvement of Mct8 in testis and sperm development in a rat

**DOI:** 10.1038/s41598-020-67594-2

**Published:** 2020-07-07

**Authors:** Hee Sook Bae, Yun-Kyeong Jin, Sangwoo Ham, Hee Kyoung Kim, Hyejung Shin, Gyu-bon Cho, Kyu Jun Lee, Hohyeon Lee, Kyeong-Min Kim, Ok-Jae Koo, Goo Jang, Jung Min Lee, Jae Young Lee

**Affiliations:** 1 ToolGen, Inc., #1204, Byucksan Digital Valley 6-cha, Seoul, South Korea; 2 Laboratory of Theriogenology, Department of Veterinary Clinical Science, College of Veterinary Medicine, Seoul National University, Seoul, South Korea; 3 School of Life Science, Handong Global University, Pohang, 37554 South Korea

**Keywords:** Metabolism, Reproductive biology

## Abstract

Thyroid hormone (TH) has long been believed to play a minor role in male reproduction. However, evidences from experimental model of thyrotoxicosis or hypothyroidism suggests its role in spermatogenesis. Cellular action of TH requires membrane transport via specific transporters such as monocarboxylate transporter 8 (MCT8). *SLC16A2* (encodes for MCT8) inactivating mutation in humans can lead to Allan-Herndon Dudley-syndrome, a X-linked psychomotor and growth retardation. These patients present cryptorchidism which suggests a role of MCT8 during spermatogenesis. In this study, we found that Mct8 is highly expressed during early postnatal development and decreases its expression in the adulthood of testis of wild-type male rats. Histological analysis revealed that spermatogonia largely lacks MCT8 expression while spermatocytes and maturing spermatids highly express MCT8. To further understand the role of Mct8 during spermatogenesis, we generated *Slc16a2* (encodes MCT8) knockout rats using CRISPR/Cas9. Serum THs (T3 and T4) level were significantly altered in *Slc16a2* knockout rats when compared to wild-type littermates during early to late postnatal development. Unlike *Slc16a2* knockout mice, *Slc16a2* knockout rats showed growth delay during early to late postnatal development. In adult Slc16a2 knockout rats, we observed reduced sperm motility and viability. Collectively, our data unveil a functional involvement of MCT8 in spermatogenesis, underscoring the importance of TH signaling and action during spermatogenesis.

## Introduction

Thyroid hormones (THs) are well known to play a crucial role in the development of the central nervous system and in the regulation of cellular differentiation and metabolism^1,2^. Both prohormone, thyroxine (T4) and relatively minimal amount of genomically active tri-iodothyronine (T3) secreted from thyroid gland can be actively transported into the cells via specific transporters expressed at the plasma membrane^1^. These include monocarboxylate transporter 8 and 10 (MCT8 and MCT10), organic anion-transporting polypeptide 1c1 (OATP1C1) and L-type amino acid transporter 1 and 2 (LAT1 and LAT2)^3^. All of these transporters transport aromatic amino acids except MCT8 which only transports THs, positioning it as the most specific TH transporter^4^.

Male reproductive organs such as testis and epididymis were long thought to be unresponsive organs for THs. However increasing evidences suggest for their potential role in these organs.

Molecular machineries that can exert cellular TH entry to its genomic action were all shown to be expressed in testis^5^. For instance, specific nuclear receptors for THs which is required to exert gene transcription mediated by THs are shown to be expressed in both rodent and human testis^6,7^. Furthermore, TH transporters and deiodinases were shown to be expressed in testis^5^.

The potential impact of TH in male reproductive organs development is manifested in animal models of hypo- or hyperthyroidism which resulted in substantial alteration in physiology of testis development^8^. TH action is not only dependent on the serum concentrations of the hormones, but also on tissue-specific cellular TH transporters, which can regulate local TH availability and elicit cell-specific developmental events. Human loss-of-function mutation of MCT8, Allan-Herndon-Dudley syndrome (AHDS) shows cryptorchidism (undescended testes), demonstrating functional significance of TH transporter in testicular development^9,10^. In an attempt to recapitulate phenotypes related to AHDS, mice lacking Mct8 were generated^11,12^. Although, serum TH hormone levels were found to be altered as observed in AHDS patients, no phenotypes related to abnormal testis development were reported^11,12^.

Because rat is more physiologically relevant to human than mouse^13^ and represented phenotypes of human which are not observed in the mouse counterpart^14^, we sought to develop a novel rat model of Mct8 deficiency using CRISPR/Cas9 gene-editing. Our data shows that, unlike mice lacking Mct8, mutant rat showed significant growth retardation and altered spermatogenesis, which may implicate the potential importance of proper TH transport for mammalian gonad development.

## Results

### Mct8 is expressed on rat testis

**Figure 1:**
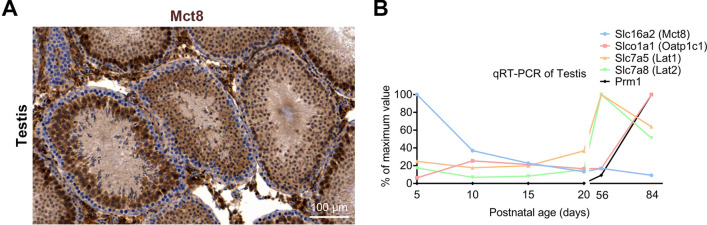
TH transporters expression in the rat testis during its development. (**A**) Histological examination of Mct8 expression in the wild-type rat testis at postnatal day 56. Note that the Mct8 is absent in immature germ cells (spermatogonium) whereas it is expressed in maturing germ cells (spermatocytes). (**B**) Gene expression analysis of TH transporters in rat testis during development. The data are expressed relative to highest expression of each genes tested. *n* = 3.

Given potential impact of TH levels on gonadal development^5^, we first immunostained testis section from wild-type adult male rat (p50) with anti-Mct8 and found the expression of Mct8 primarily in maturing spermatids whereas spermatogonia largely lacks its expression (Fig. 1A, Supplementray Fig. 1). Furthermore we also immunostained cauda epididymis and found Mct8 is localized to epithelium and spermatozoa (Supplementary Fig. 2). We then seek to identify the expression pattern of known TH transporters previously shown to be expressed in rodent testis^15,16^ (for review see^17^) throughout developmental stages. For this, we performed qRT-PCR-based gene expression analysis of transcripts of TH transporters, *Slc16a2* (Mct8), *Slco1a1* (Oatp1c1), *Slc7a5* (Lat1) and *Slc7a8* (Lat2) at postnatal day 5, 10, 15, 20, 56 and 84 (p5, p10, p15, p20, p56, p84). Prm1 (Protamine, which is known to be expressed on in the adult testis) was included as internal control. In line with previous observation in mouse^16^, Mct8 is highly regulated when compared to other TH transporters during early developmental stage (from p5 to p15) (Fig. 1B and Supplementary Fig. 3). As Sertoli, Leydig and Germ cell proliferation were reported to occur until p15 during rodent testis development^5^ and as Mct8 is the most efficient and specific TH transporter^3^, highly regulated expression of Mct8 during early testis development indicates the importance of TH action for testicular cell proliferation. However, the gene expressions for other TH transporters such as Oatp1c1, Lat1 and Lat2 were found to sharply increase during adulthood which may suggest for their role during adulthood.

**Figure 2:**
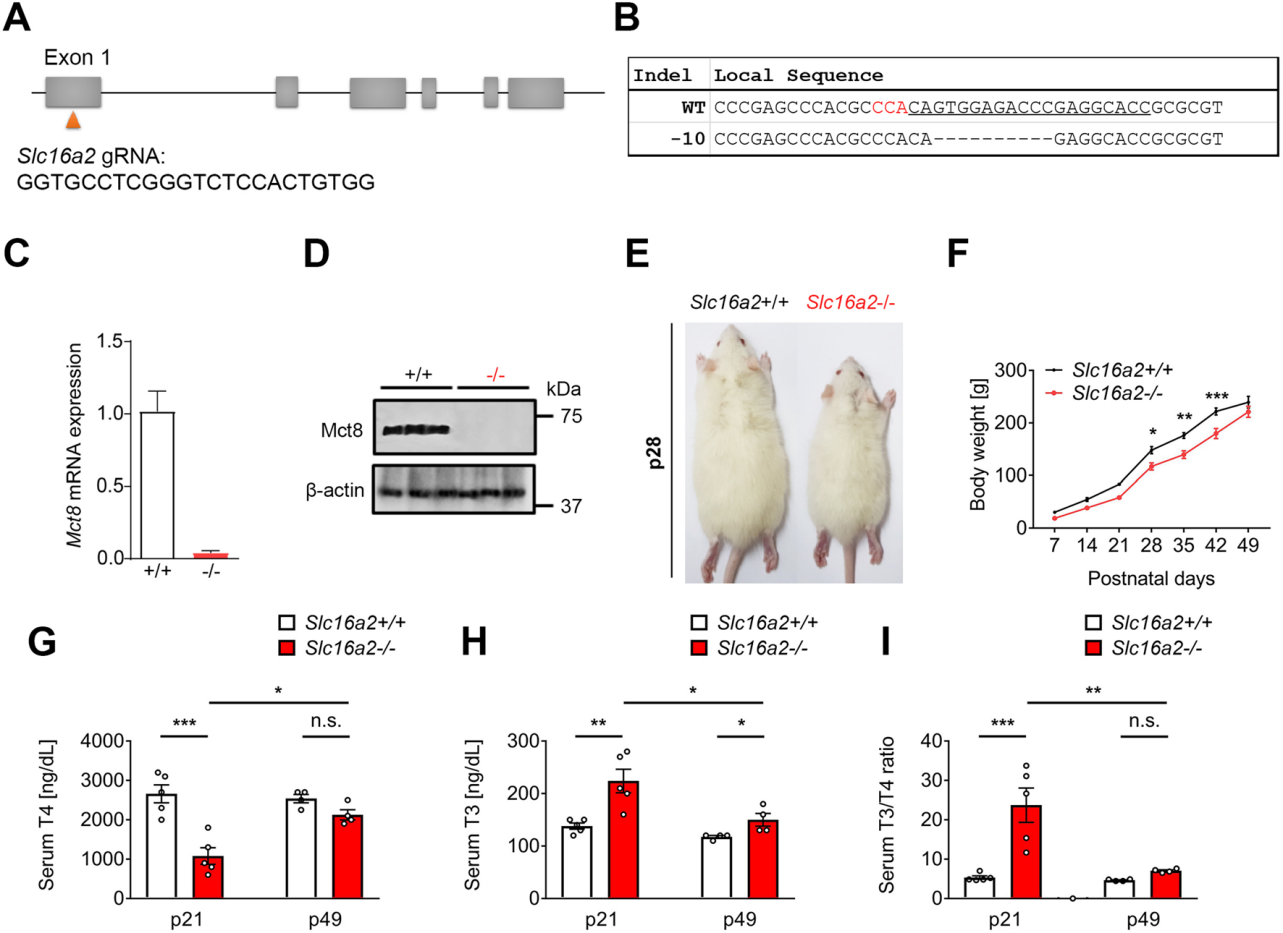
Transient growth retardation associated with altered serum TH levels in *Slc16a2*−/− rats generated using CRISPR/Cas9. (**A**) Schematic representation of the targeting site at rat *Slc16a2* loci. Exon 1 of rat *Slc16a2* was targeted using specific CRISPR gRNA shown below. (**B**) Targeted deep-sequencing reads of the mutant allele from F0 founder. The gRNA-targeting sequences are underlined and the PAM sequence is highlighted in red. The deletions are indicated as. (**C**) qRT-PCR analysis of testes of F3 founders of *Slc16a2*+/+ and *Slc16a2*−/− (*n* = 5 for each genotypes). (**D**) Immunoblot analysis of testes of F3 founders of *Slc16a2*+/+ and *Slc16a2*−/−. (**E**) A representative image of *Slc16a2*+/+ and *Slc16a2*−/− male rats at postnatal day 28, showing growth retardation phenotype in *Slc16a2*−/− male rat. (**F**) The growth curve of *Slc16a2*+/+ and *Slc16a2*−/− male rats showing a transient growth retardation of *Slc16a2*−/− from p28-42. (**G**) Serum T4 concentrations of *Slc16a2*+/+ and *Slc16a2*−/− male rats were determined at p21 and p49. *Slc16a2*−/− rats. (**H**) Serum T3 concentrations of *Slc16a2*+/+ and *Slc16a2*−/− male rats were determined at p21 and p49. (**I**) Serum T3/T4 ratio of *Slc16a2*+/+ and *Slc16a2*−/− male rats at p21 and p49. *n* = 5, *P* < 0.05;*, *P* < 0.01;**, *P* < 0.001;***.

**Figure 3:**
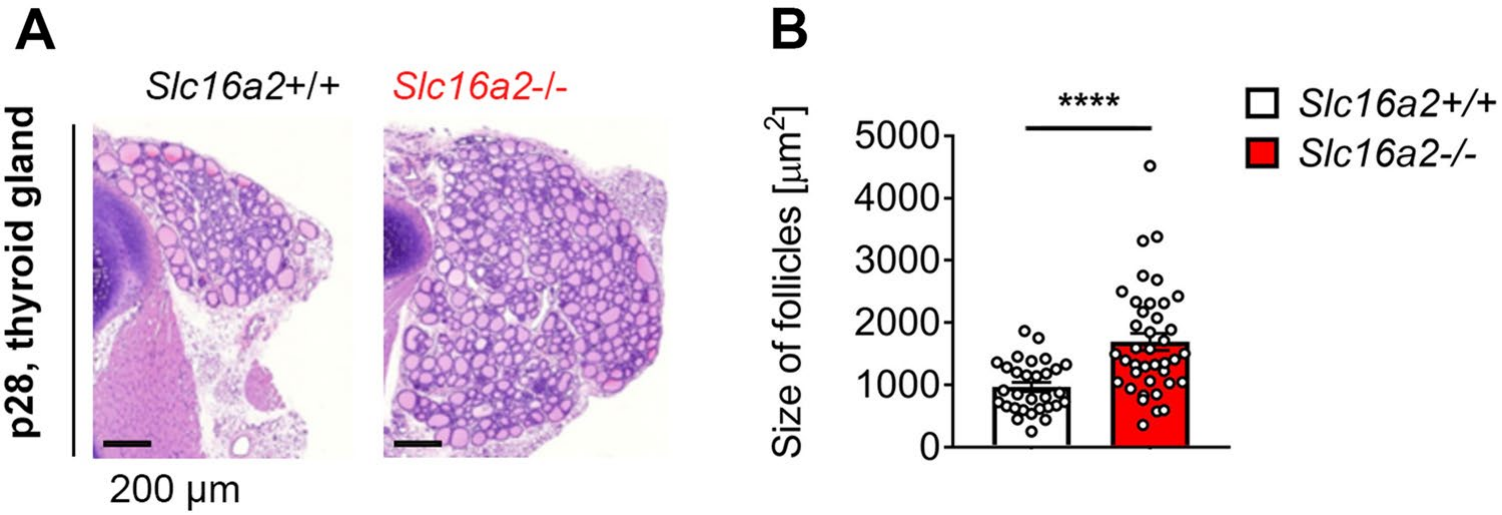
Histological examination of *Slc16a2*+/+ and *Slc16a2*−/− male rat thyroid glands. (**A**) Schematic representation of H&E-stained sections from thyroid glands of p28 *Slc16a2*+/+ and *Slc16a2*−/− male rats. (**B**) Morphometric analysis of thyroid gland sections showing the size of follicle. *n* = 5, *P* < 0.0001;****.

### Generation of Mct8 deficient (*Slc16a2* −/−) rats

To investigate the potential impact of Mct8 in testicular development, we electroporated Cas9 protein and CRISPR gRNA targeting exon 1 of rat *Slc16a2* (Fig. 2A) into pronuclear-stage embryos of Sprague–Dawley rats. Upon embryo transfer of electroporated embryos to pseudo-pregnant foster mothers, we obtained mutant pups that were found to have 10 base pairs deletion analysed by targeted deep sequencing (− 10; Fig. 2B; Supplementary Table 1). This − 10 deletion mutation in exon 1 is expected to cause premature stop codon which can induce knockout of *Slc16a2* and as expected, mRNA of *Slc16a2* was barely detectable in the testis of *Slc16a2*−/− rats (Fig. 2C). We also performed western blot analysis and further validated undetectable expression of Mct8 in Slc16a2−/− rat testis (Fig. 2D). For maintenance of the colony, we crossed *Slc16a2*+/+ male rats with *Slc16a2* ± female rats. The genotypes of offspring derived from this mating was consistent with Mendelian inheritance (Supplementary Table 2). To evaluate the fertility of the mutant male rats, we crossed *Slc16a2*−/− male rats with *Slc16a2*+/+ female rats and normal litter size was obtained when compared with *Slc16a2*+/+ male rats (Supplementary Fig. 4). To further examine fertilization ability, we performed in vitro fertilization test by inseminating the oocytes from *Slc16a2*+/+ rats with the sperm from *Slc16a2*−/− and *Slc16a2*+/+ rats. This revealed a lower fertilizing ability of *Slc16a2*−/− rat sperm (lower number of embryos developed into two-cells; Supplementary Table 3). These indicate that even though lower fertilization rate was observed in *Slc16a2*−/− rat sperm, once successfully, these embryos can develop normally. For further phenotypic analysis described throught this manuscript, F3 founders of *Slc16a2*+/+ and *Slc16a2*−/− were utilized.

**Figure 4:**
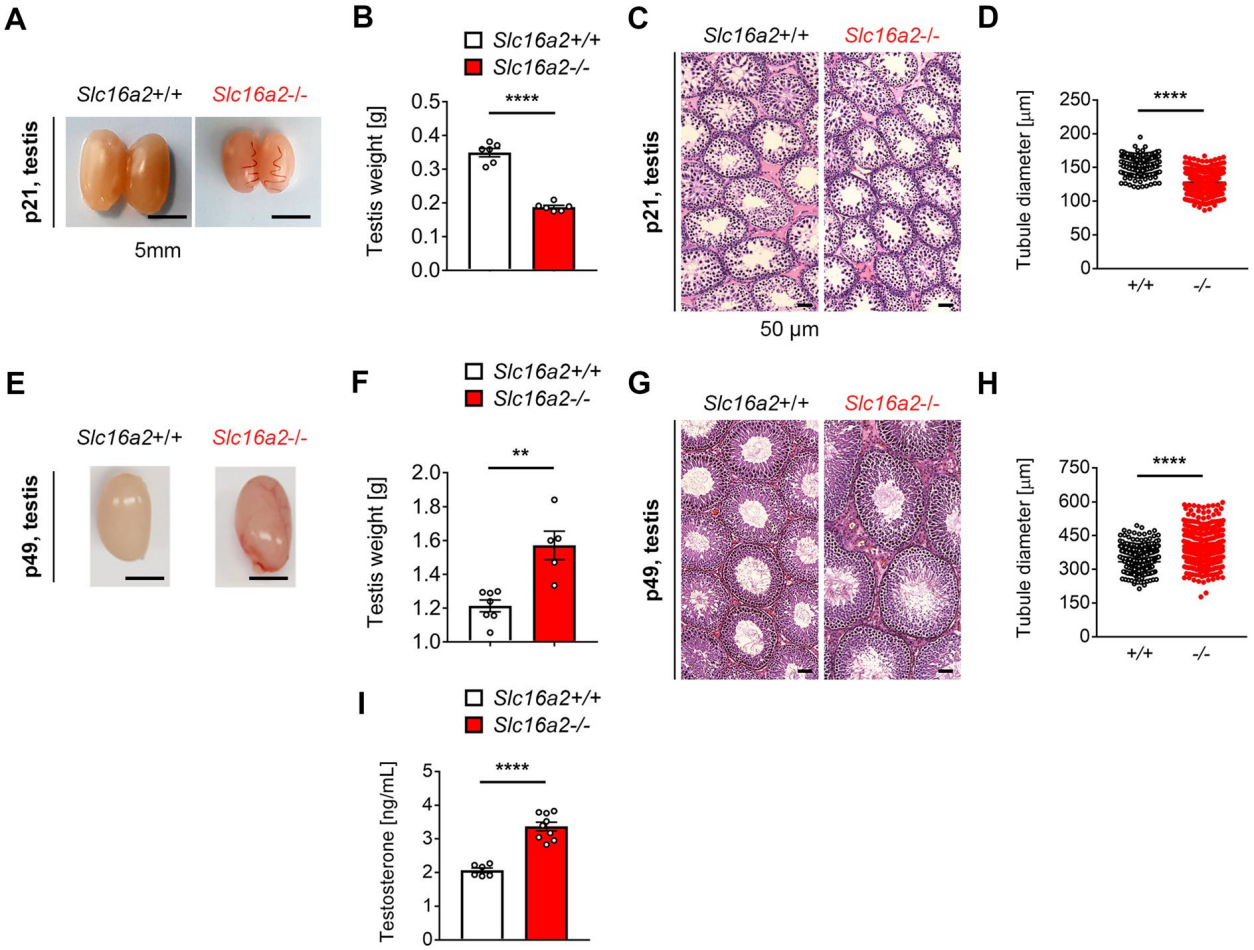
Altered testis development of *Slc16a2*−/− rat. (**A**) Representative images of *Slc16a2*+/+ and *Slc16a2*−/− rats at p21. (**B**) Testis weights of *Slc16a2*+/+ and *Slc16a2*−/− rats at p21. *n* = 6 for each genotypes. (**C**) Representative images of H&E stained sections of *Slc16a2*+/+ and *Slc16a2*−/− rats at p21. (**D**) Diameter of seminiferous tubules of *Slc16a2*+/+ and *Slc16a2*−/− rats at p21 *n* = 4 for each genotypes. (**E**) Representative image of *Slc16a2*+/+ and *Slc16a2*−/− rats at p49. (**F**) Testis weights of *Slc16a2*+/+ and *Slc16a2*−/− rats at p49. *n* = 7 for *Slc16a2*+/+ and *n* = 5 for *Slc16a2*−/−. (**G**) Representative images of H&E stained testis sections of *Slc16a2*+/+ and *Slc16a2*−/− rats at p49. (**H**) Diameter of seminiferous tubules of *Slc16a2*+/+ and *Slc16a2*−/− rats at p49. *n* = 4 for each genotypes. (**I**) Serum testosterone levels of *Slc16a2*+/+ and *Slc16a2*−/− male rats at p49. *n* = 6 for *Slc16a2*+/+ and *n* = 9 for *Slc16a2*−/−, *P* < 0.01;**, *P* < 0.001;*** *P* < 0.0001;****.

### Reduced growth rate along with thyroid dysfunction of *Slc16a2*− /− rats

Unlike *Slc16a2*−/− mice^12,15,18^, *Slc16a2*−/− rats showed growth delay with visibly smaller body size observed from postnatal days 28 until 42 (Fig. 2E) and this difference was alleviated from postnatal day 49 (Fig. 2F). We next analyzed serum TH status of *Slc16a2*−/− male and their wild-type littermates at p21 and p49. In *Slc16a2*−/− rats, serum T4 levels were significantly decreased at p21, however, there were no significant differences between *Slc16a2*−/− and *Slc16a2*+/+ serum T4 levels at p49. (Fig. 2G). On the other hand, the serum T3 levels of *Slc16a2*−/− was found to be significantly increased when compared to *Slc16a2*+/+ rats at p21 and this difference was lessened at p49 (Fig. 2H). Overall, there was 4.47 fold increase in the mean T3/T4 ratio in the *Slc16a2*−/− male rats at p21 which was partially normalized by p49 (1.51 fold increase in the mean serum T3/T4 ratio in *Slc16a2*−/− when compared to *Slc16a2*+/+ rats) (Fig. 2I). Thus, these results indicate that in *Slc16a2*−/− male rats, serum hyperthyroidism (increased serum T3 level) during p21 to p42 may have caused hypermetabolism which caused reduced growth rate of this mutant.

### Increased thyroid gland follicular size in *Slc16a2*− /− rats

Next, we addressed the consequences of Mct8 deficiency on thyroid gland morphology. For this, we stained thyroid gland sections with H&E from both *Slc16a2*+/+ and *Slc16a2*−/− rat males at p28. Similar to *Slc16a2* −/− mice, *Slc16a2*−/− rats showed increased thyroid gland whole follicular area (Fig. 3A,B). Furthermore, significantly increased size of follicles in thyroid gland was found in *Slc16a2*−/− when compared to *Slc16a2*+/+ male rats. This may suggest that as reported in *Slc16a2*−/− mice^18,19^, thyroidal TH concentration may have increased in *Slc16a2*−/− male rats which was reflected as increase in follicle size.

### Testis maldevelopment in *Slc16a2*−/− rats

To address whether *Slc16a2*−/− male rats recapitulate abnormal testicular development as reported in AHDS patients, we first examined gross morphology of testis from *Slc16a2*+/+ and *Slc16a2*−/− rats. We found that both the size (Fig. 4A) and weight (Fig. 4B) of the testes of *Slc16a2*−/− rats were reduced when compared with *Slc16a2*+/+ rats at p21. We then performed histological analysis of H&E stained sections from testes of *Slc16a2*−/− and *Slc16a2*+/+ rats (Fig. 4C). Although no obvious defects were identified, we found that *Slc16a2*−/− rats showed reduced diameters of round seminiferous tubules when compared with *Slc16a2*+/+ rats (Fig. 4D). We also performed similar analysis at p49, a timepoint when testis are fully mature during normal rat development. On the contrary to p21, we identified increased both the size (Fig. 4E) and weight of the testes of *Slc16a2*−/− rats (Fig. 4F) when compared with *Slc16a2*+/+ rats which is similar observation as reported in *Slc16a2*−/− mice^16^. As neonatal alteration of TH can lead to central abnormalities in the hypothalamic-pituitary-thyroid axis, we analysed serum testosterone level at p49. This analysis revealed a significant increase in serum testosterone level in *Slc16a2*−/− rats when compared to *Slc16a2*+/+ rats, which may have contributed to enlargement of testis size in *Slc16a2*−/− rats. Furthermore, as the size of testis was reported to be highly dependent on the number of Sertoli cells^20^, we counted the number of Sertoli cells per seminiferous tubules at p28 and p56 by immunostaining against Vimentin, a cytoplasmic Sertoli cell marker^21^. We found that, the number of Sertoli cells per seminiferous tubules was reduced at p28, whereas increased at p56 in *Slc16a2*−/− rats when compared with *Slc16a2*+/+ rats (Supplementary Fig. 5). These results indicates that the local hypothyroidism due to Mct8 deficiency in *Slc16a2*−/− rats may cause delay in Sertoli cell differentiation and prolonged proliferation phase.

**Figure 5:**
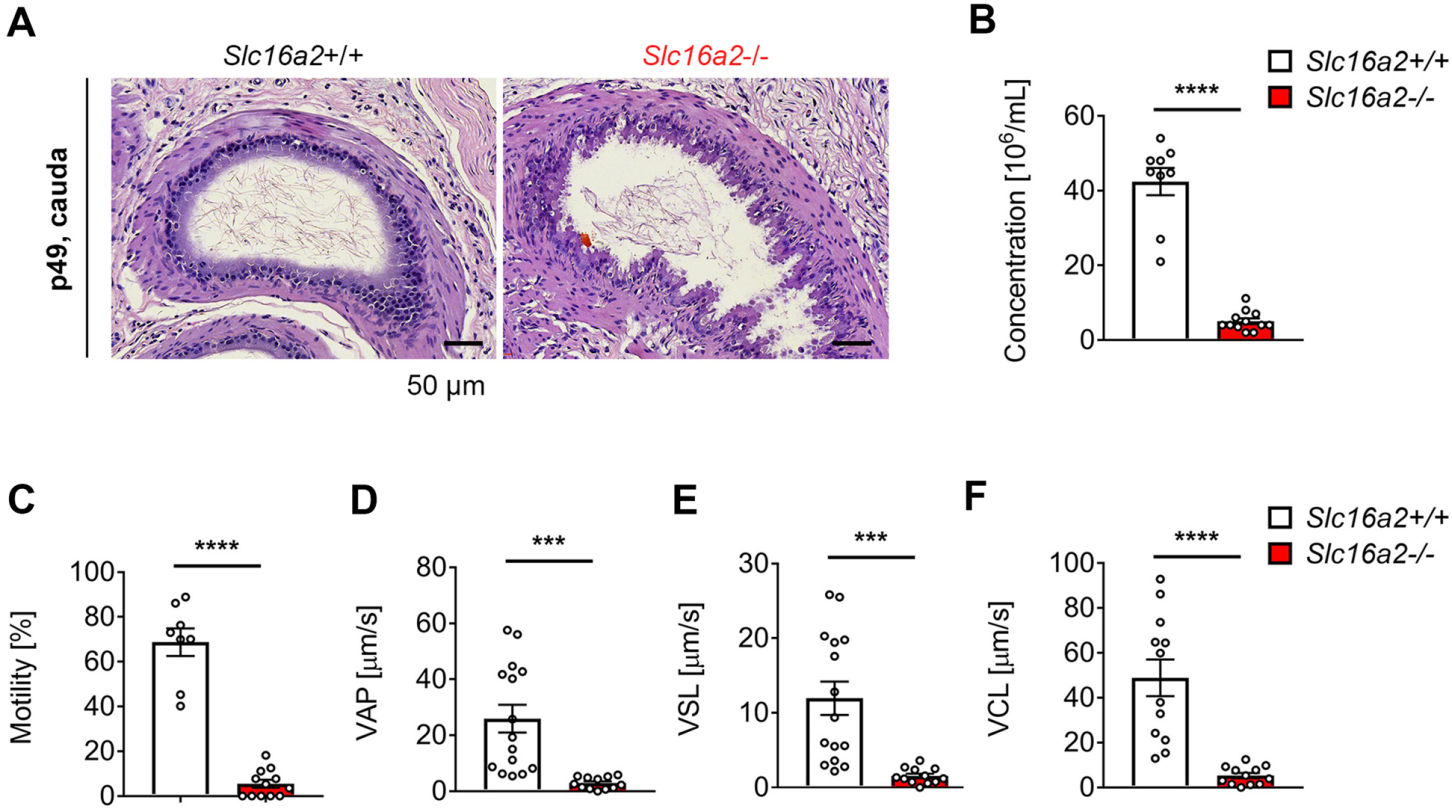
Altered spermiogenesis of *Slc16a2*−/− rat. (**A**) Representative images of H&E stained cauda epididymis sections of *Slc16a2*+/+ and *Slc16a2*−/− rats at p49. Scale bar = 50 μm. (**B–E**) CASA analysis of spermatozoa of *Slc16a2*+/+ and *Slc16a2*−/− rats at p49. (**B**) Spermatozoa concentration; (**C**) spermatozoa motility; (**D**) spermatozoa average path velocity (VAP); (**E**) spermatozoa straight line velocity (VSL); (**F**) spermatozoa curvilinear velocity (VCL). *n* = 12, *P* < 0.001;*** *P* < 0.0001;****.

### Impaired spermiogenesis in Slc16a2− /− rats

To further investigate the impact of lacking Mct8 in male reproductive biology, we investigated spermiogenesis. For this, we prepared H&E stained sections from cauda epididymis from *Slc16a2*+/+ and *Slc16a2*−/− rats. We identified abnormal spermatozoa in Slc16a2−/− rats at p49. Furthermore, the sperm density was reduced in *Slc16a2*−/− when compared with *Slc16a2*+/+ rats at p49 (Fig. 5A and Supplementary Fig. 6). These were also found in corpus epididymis where sperm density was found reduced in *Slc16a2*−/− when compared with *Slc16a2*+/+ rats (Supplementary Fig. 6). Moreover, caudal epididymal spermatozoa imaging revealed that abnormal sperm morphology was found in *Slc16a2*−/− when compared with *Slc16a2*+/+ rats (Supplementary Fig. 7). Furthermore, epididymal epithelial cells seemed abnormal in *Slc16a2*−/− when compared with *Slc16a2*+/+ rats (Fig. 5A). To further characterize potential deficit in spermiogenesis, we performed computer-assisted semen analysis (CASA) to examine sperm concentration and motility in *Slc16a2*−/− rats at p49. We found that the number of spermatozoa was reduced in *Slc16a2*−/− rats when compared with *Slc16a2*+/+ rats (Fig. 5B). Furthermore, sperm motility of *Slc16a2*−/− rats were decreased when compared with *Slc16a2*+/+ rats (Fig. 5C, Supplementary Video 1 and 2). All other parameters of CASA imaging, including average path velocity (VAP), straight line velocity (VSL) and curvilinear velocity (VCL) (Fig. 5D–F).

Together, these results indicate that Mct8 deficiency in male rats can affect testis development and spermiogenesis.

## Discussion

It has been well established that TH level influences testis development^5^. TH action in local tissues and cells can be regulated by specific TH transporters such as Mct8^3^. The potential importance of Mct8 in testis development was suggested from the patients with AHDS where cryptorchidism^4,9^. Furthermore, we have found that Mct8 is expressed throughout early to late postnatal development as a dominant TH transporter, indicating potential impact of this transporter in testis development (Fig. 1). Interestingly, developmental expression pattern of *Slc16a2* is somewhat in line with mouse developmental expression pattern of *Thra*, a nuclear TH receptor highly expressed in Sertoli cells^5^. One can expect a potential important role of Mct8 in TH transport in Sertoli cells.

To study the potential impact of Mct8 deficiency in testis development, we generated the rat model of Mct8 deficiency utilizing CRISPR/Cas9 technology (Fig. 2A,B). As genome and physiology of rats has been shown to be closer to humans^22–24^, we selected rats rather than mice to study the role of Mct8 in testis development. In fact, we found a transient growth delay in *Slc16a2*−/− rats (Fig. 2D,E) which was not found in *Slc16a2*−/− mice^11,12^. Slow growth rate during early to late postnatal development (p21-42) in *Slc16a2*−/− rats seemed to be associated with serum hyperthyroidism as during these timepoints, we found a significant increase in serum T3 level at early postnatal development (p21). This increased level of serum T3 were normalized (similar to *Slc16a2*+/+ level) by p49 (Fig. 2G,H) which correlated with normalization of body weight of *Slc16a2*−/− (similar to *Slc16a2*+/+ rats) by p49 (Fig. 2E). These results may indicate an utility of rats as a model to study thyroid-related metabolism.

In support of transient reduced growth rate during early to late postnatal development, the dramatic reduction in testis size were found in *Slc16a2*−/− rats at p21, which was correlated with reduced seminiferous tubule size (Fig. 4A–D). This observation is in line with previous data where reduced testis size were caused by serum hyperthyroidism^25,26^. On the other hand, we found a larger testis of *Slc16a2*−/− rats at p49 with increased seminiferous tubule size (Fig. 4E–H). This effect was supported by the Sertoli cell numbers in different stage of development. As active thyroid hormone, T3 was found to play a key role in Sertoli cell cycle exit and differentiation^8^, local testis hypothyroidism due to Mct8 deficiency may prolonged Sertoli cell proliferation.

Other potential effect that alteration of TH may have is the development of the blood-testis barrier as literatures suggested that the expression of connexin 43 is regulated by TH^27,28^. Furthermore, hyperthyroidism was shown to elevate the number of Leydig cells^29^. Therefore, in future study, it will be interesting to investigate the blood-testis barrier and Leydig cells in *Slc16a2*−/− rats.

Other than testis development, we identified that Mct8 deficiency led to partially disrupted epididymal epithelium, which may suggest a potential role of TH in epididymal epithelial cell maintenance. We found the expression of Mct8 in epididymal epithelial cells and previous literature reported the expression of nuclear TH receptor (TR) in both cytosol and nucleus of rat epididymal epithelial cells. These suggest a potential role of TH in both genomic and non-genomic way in epididymal epithelial cells^30^. Therefore, in Mct8 deficient scenario, there would be reduced TH transport into epididymal epithelial cells, which may have caused abnormal cellular physiology. Further studies are required to identify exact roles of TH on epididymal epithelial cells.

We have performed immunohistochemistry with anti-Mct8 antibody and found that Mct8 is expressed in epididymal epithelial cells (Supplementary Fig. 1). Epididymal spermatozoa seems also express Mct8 (Supplementary Fig. 1). As nuclear thyroid hormone receptor (TR) was found to be expressed on both cytosol and nucleus of rat epididymal epithelial cells, indicating a potential role of thyroid hormone in both genomic and non-genomic way^30^. Therefore, in Mct8 deficient scenario, there would be reduced thyroid hormone transport into epididymal epithelial cells which may caused normal cellular physiology.

As previous literatures suggested that thyroid hormones are required for proper functioning sperm cytoskeleton^31–33^, insufficient TH uptake by sperms in Slc16a2−/− rats would cause motility and shape abnormalities. Indeed, in our *Slc16a2*−/− rats, we found a reduction in number of sperms in epididymis and a significant changes in motility parameters from the CASA analysis. As alteration in TH levels in human were shown to adversely affect semen quality by compromising progressive sperm^31^, our results provide a direct evidence that alteration of TH levels by Mct8 deficiency affects sperm maturation in the epididymis.

Collectively, our results demonstrate that Mct8 plays an important role in TH action in the testis development and sperm maturation and motility. The *Slc16a2*−/− rat presented in this study can be a valuable model for studying the developmental and functional abnormalities in different tissues due to the alteration of THs, which only testis were partially explored in this study. As altered TH levels can affect other important cellular physiology as observed in patients with AHDS, it would be interesting to find out whether the present *Slc16a2*−/− rat model recapitulate any of these phenotypes.

## Methods

### Animals and ethics statement

Wild-type Sprague–Dawley (SD) female rats (7–13 weeks old) were purchased from Orient Bio (Korea). Rats were kept in animal facility (Seoul National University, Veterinary College). Standard water and food were supplied ad libitum (Daehan Bio Link). Animal room was climate controlled to provide temperatures of 22–25 °C on a 12 h light/dark cycle. All the experimental procedures as described below were performed in accordance with the IACUC guidance and regulations approved by the Seoul National University Institutional Animal Care and Use Committee (SNU-160719–2-7).

### Generation of *Slc16a2*− /− rat

We generated *Slc16a2* knockout rats by CRISPR/Cas9 genome editing. Genome Editor electroporator and LF501PT1-10 platinum plate electrode (length: 10 mm, width: 3 mm, height: 0.5 mm, gap: 1 mm) (BEX Co. Ltd., Tokyo, Japan) were used for electroporation.

The electrode was connected to the electroporator and was set under a stereoscopic microscope. 30–40 zygotes prepared by natural breeding (NB) electroporation at one time. The electroporation conditions were 30 V (3 ms ON + 97 ms OFF) × 7 times. Subsequently, the eggs were collected from the electrode chamber and subjected to four washes with M2 medium (Sigma) followed by four washes with mR1ECM medium(ARK Resource). After the incubation with mR1ECM medium(ARK Resource) at 37 °C and 5% CO2, the eggs were allowed to develop to the two-cell stage and then transferred into pseudopregnant females.

### Immunoblot analysis

Tissues were homogenized and lysed with Pierce RIPA buffer (25 mM Tris–HCl (pH 7.6), 150 mM NaCl, 1% NP-40, 1% sodium deoxycholate, 0.1% SDS, Thermo Scientific, Cat # 89,900) for 30 min on ice. Tissue lysates were prepared by centrifugation (13,000 × rpm at 4 °C for 30 min). Protein concentration was determined using Pierce BCA protein assay kit (Thermo Scientific, cat # 23,225). Equal amounts of protein (20 μg) were resolved on 4 to 15% SDS-PAGE and transferred to nitrocellulose membrane (Bio-Rad, Cat # 1,620,115). After washing with TBS-T, the membranes were blocked with 2.5% skim milk for 30 min and incubated with appropriate primary antibodies Anti-MCT8 (Abcam, ab192828) and Anti-β-actin (Sigma, A2228). The membrane was washed then primary antibodies were detected with an HRP-conjugated secondary antibody.

### qRT-PCR analysis

RNA was isolated from testis by using RNeasy Mini Kit (Qiagen) according to the manufacturer’s instruction and treated with DNase-I to remove genomic DNA. The concentrations of total RNA were measured by Nanodrop ND-1000 spectrophotometer v3.7 (Thermo Specific) and complementary DNA (cDNA) was synthesized from 1 μg of total RNA using High-Capacity cDNA Reverse Transcription Kit (Applied Biosystems) according to the manufacturer’s instructions. Real-Time Quantitative Reverse Transcription Polymerase Chain Reaction (qRT-PCR) was performed with 5–20 ng of cDNA template, 2 × Taqman Gene expression master mix and 20 × Taqman probes (Life technologies) on ABI Prism 7900HT Sequence detection system (Applied Biosystems). *Gapdh* served as an endogenous standard control. The qRT-PCR thermo-cycling reaction was 1 cycle at 50 °C for 2 min, 1 cycle at 95 °C for 10 min and 40 cycles of 95 °C for 15 s, then 60 °C for 1 min.

Accession number of Taqman probes used for qRT-PCR.

**Table:** 

Target Gene	Taqman Gene Expression Assay
*Gapdh*	Rn01775763_g1
*Slc16a2*	Rn00596041_m1
*Slco1c1*	Rn00584891_m1
*Slc7a5*	Rn00569313_m1
*Slc7a8*	Rn00584909_m1
*Prm1*	Rn02345725_g1

### Measurement of serum TH, Testosterone

Blood was collected in SST (Serum separate tube) tubes by abdominal vein puncture and immediately centrifuged at 3,000 rpm for 10 min. The serum samples were stored at − 80 °C until further analysis. T3, T4 and TSH concentrations in serum were measured using an electrochemiluminescence immunoassay (Elecsys, Roche, Germany). Testosterone concentrations in serum were measured using an Enzyme-Linked Immunosorbent Assay (Testosterone Rat/Mouse ELISA, Calbiotech, USA).

### Histology

Testes were fixed in Bouin’s solution for 2 h (fetal and neonatal testes) or overnight (adult testes), briefly washed in 70% ethanol, cut transversally in 2 halves and paraffin embedded for sectioning at the cut area. Hematoxylin and eosin (H&E) staining, as well as immunohistochemistry staining for the The diameter of a seminiferous tubule was defined as the shortest distance between two parallel tangent lines of the outer edge of the tubule. Testis sections of 4 mice/group were obtained by optical microscopy using an Olympus IX51 camera × 10-microm eyepiece coupled with an × 10 objective glass (Olympus Japan). The seminiferous tubule diameter was measured using the QCapture Pro software (version 6.0; QImaging). ~ 200 tubules were measured per animal.

Immunohistochemical staining was done using UltraVision LP Large Volume Detection System (Thermo Scientific) according to the manufacturer’s procedure. Briefly, 5-μm thick sections were deparaffinized in xylene, and rehydrated in decreasing ethanol concentrations. After antigen retrieval with Proteinase K, the sections were incubated with blocking solution (Hydrogen Peroxide Block) for 10 min at room temperature (RT). The primary antibody Anti-MCT8 (MBL, BMP031) were applied and incubated for overnight at 4 °C. After washing the primary antibody, the slides were incubated with Primary Antibody Enhancer for 20 min at RT, followed by incubation with HRP Polymer for 30 min at RT. The sections were developed using DAB (DAB + Substrate Chromogen System, DAKO) and then were counterstained with Mayer's hematoxylin.

### In vitro fertilization (IVF)

Sprague Dawley female rats (4-weeks-old) were induced to superovulate using intraperitoneal injections of PMSG on Day 1 and hCG on Day 3. On Day 4, male rats were sacrificed and the cauda epididymis were quickly removed, allowing the sperm capacitates to place in the incubator for 1 h before insemination. During this period, the superovulated female rat was sacrificed and the ampullae were quickly removed. To collect cumulus-oocyte-complex (COC), the ampullae were placed in a fertilization dish (FERTIUP PM 0.5ML-CARD MEDIUM set, KYD-005-EX). The sperm added into the fertilization dish included the COC or cumulus-free oocytes using a pipette. The fertilization dish was incubated for approximately 3 h at 37 °C. After this incubation, embryo were removed from the fertilization dish and washed 3 times with the HTF (ARK Resource Co., Ltd.). The HTF drops that covered with paraffin oil incubated for overnight at 37 °C and the number of two-cell was counted on Day 5.

### CASA

Mature male rats were sacrificed and the two epididymis were removed aseptically. Left and right epididymis were transferred to the 10–12 ml liquid paraffin in a 60 mm petri dish. A small incision around 0.5 mm was made in the caudal epididymis and 1–2 drop of sperms were transferred to pre-warmed M2 media to dilute 1:500 and maintained at 37 °C for 3 min. During this time, the dish was rotated every minute to disperse sperm. Thirty microliter sperms near the marginal drop were collected and loaded on standard count 2-chamber slides (Leja, SC 100–01-02-B). Sperm motility was analyzed by CASA and images were taken by Olympus BX 50. Samples were analysed three times at different sites.

### Statistical analysis

The GraphPad Prism software for statistical analysis. Unless stated otherwise, comparisons between more than 2 experimental groups were performed by ANOVA and Tukey’s post hoc test, whereas statistical significance between 2 groups was determined by the Student’s *t* test. *P* value smaller than 0.05 was considered statistically significant.

## Supplementary information

Supplementary file1 (DOCX 6035 kb)

Supplementary file2 (AVI 13277 kb)

Supplementary file3 (AVI 3725 kb)

## Data Availability

The raw data presented in this manuscript is available upon request to JYL (jy.lee2@toolgen.com).
