# Author Correction: CRISPR/Cas9-mediated knockout of Mct8 reveals a functional involvement of Mct8 in testis and sperm development in a rat

**DOI:** 10.1038/s41598-020-76579-0

**Published:** 2020-11-11

**Authors:** Hee Sook Bae, Yun-Kyeong Jin, Sangwoo Ham, Hee Kyoung Kim, Hyejung Shin, Gyu-bon Cho, Kyu Jun Lee, Hohyeon Lee, Kyeong-Min Kim, Ok-Jae Koo, Goo Jang, Jung Min Lee, Jae Young Lee

**Affiliations:** 1 ToolGen, Inc., #1204, Byucksan Digital Valley 6-cha, Seoul, South Korea; 2 Laboratory of Theriogenology, Department of Veterinary Clinical Science, College of Veterinary Medicine, Seoul National University, Seoul, South Korea; 3 School of Life Science, Handong Global University, Pohang, 37554 South Korea

Correction to: *Scientific Reports* 10.1038/s41598-020-67594-2, published online 07 July 2020

The original version of this Article contained an error in the title of the paper, where “CRISPR” was incorrectly given as “CRISRP”. This has now been corrected in the PDF and HTML versions of the Article.

