# Supplementary material for: CRISPR/Cas9-mediated knockout of Mct8 reveals a functional involvement of Mct8 in testis and sperm development in a rat
Source: Sci Rep. 2020 Jul 7;10:11148. doi: 10.1038/s41598-020-67594-2 (PMC7341756; doi:10.1038/s41598-020-67594-2)
Supplement: Supplementary file 1 — Supplementary file1 (DOCX 6035 kb) [file 41598_2020_67594_MOESM1_ESM.docx]

Supplementary Information for

**CRISPR/Cas9-mediated knockout of Mct8 reveals a functional involvement of Mct8 in testis and sperm development in a rat**

**Hee Sook Bae^1#^, Yun-Kyeong Jin^2#^, Sangwoo Ham^1^, Hee Kyoung Kim^2^, Hyejung Shin^1^, Gyu-bon Cho^1^, Kyu Jun Lee^1^, Hohyeon Lee^1^, Kyeong-Min Kim^2^, Ok-Jae Koo^1^, Goo Jang^2^, Jung Min Lee^1,3^ , Jae Young Lee^1*^**

**Supplementary Tables**

|  | **Number of embryos electroporated** | **Embryos transferred (recipients)** | **Newborns(n)** | **KO/offspring(%)** |
| --- | --- | --- | --- | --- |
| Slc16a2 sgRNA | 139 | 50(1) | 7 | 6/7(85.7) |

**Supplementary Table 1. CRISPR/Cas9-mediated Slc16a2-/- rat generation**

Cas9 protein, Slc16a2 gRNA were electroporated into fertilized rat embryos. Two-cell embryos were then transferred into pseudopregnant female rats and newborns were genotypes by targeted deep-sequencing to evaluate knockout (KO)

| **Male newborn (n)** | **Female newborn (n)** | **Male +/+** | **Male -/-** | **Female +/+** | **Female +/-** |
| --- | --- | --- | --- | --- | --- |
| 5 | 6 | 2 | 3 | 4 | 2 |
| 6 | 6 | 3 | 3 | 2 | 4 |
| 3 | 7 | 2 | 1 | 1 | 6 |
| 4 | 4 | 3 | 1 | 2 | 2 |
| 5 | 4 | 2 | 3 | 3 | 1 |
| 2 | 11 | 1 | 1 | 9 | 2 |
| 6 | 5 | 0 | 6 | 4 | 1 |
| 5 | 4 | 3 | 2 | 3 | 1 |
| 6 | 8 | 2 | 4 | 7 | 1 |
| 10 | 3 | 7 | 3 | 1 | 2 |
| 3 | 11 | 0 | 3 | 7 | 3 |

**Supplementary Table 2. Mendelian inheritance in the offspring of Slc16a2+/+ (male) x Slc16a2+/- (female) breeding pair**

| Genotype | Trial # | No. of oocytes inseminated | No. of embryos developed to two-cell | No. of two-cell % | Mean ± SEM |
| --- | --- | --- | --- | --- | --- |
| Slc16a2+/+ | 1 | 170 | 41 | 24.1 | 20.0±2.21 |
|  | 2 | 170 | 33 | 19.4 |  |
|  | 3 | 285 | 47 | 16.5 |  |
| Slc16a2-/- | 1 | 238 | 22 | 9.2 | 7.13±1.21 |
|  | 2 | 152 | 11 | 7.2 |  |
|  | 3 | 261 | 13 | 5.0 |  |

*SEM = Standard error of the mean

**Supplementary Table 3. Success rates of fertilization and development of two-cell in IVF**

**Supplementary figures**

**
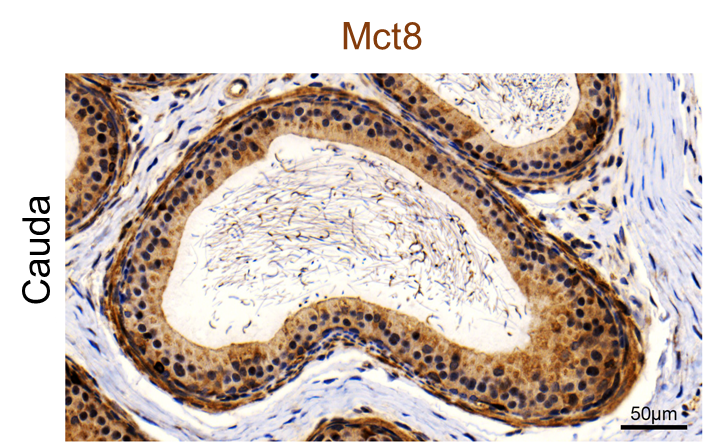
**

**Supplementary Figure 1. Mct8 expression in the rat cauda epididymis (Related to Figure 1)**

Histological examination of Mct8 expression in the wild-type rat cauda epididymis at postnatal day 56. Note that Mct8 is expressed in epithelium and spermatozoa


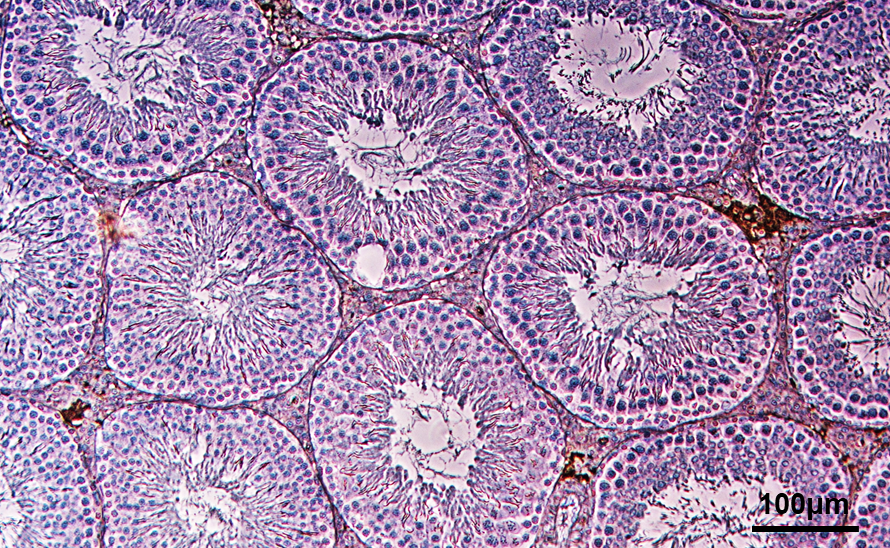


**Supplementary Figure 2. Negative control image from *Slc16a2*+/+ male rat testis (Related to Figure 1)**

Representative negative control (without primary antibody) slide from *Slc16a2*+/+ male rat testis.

**
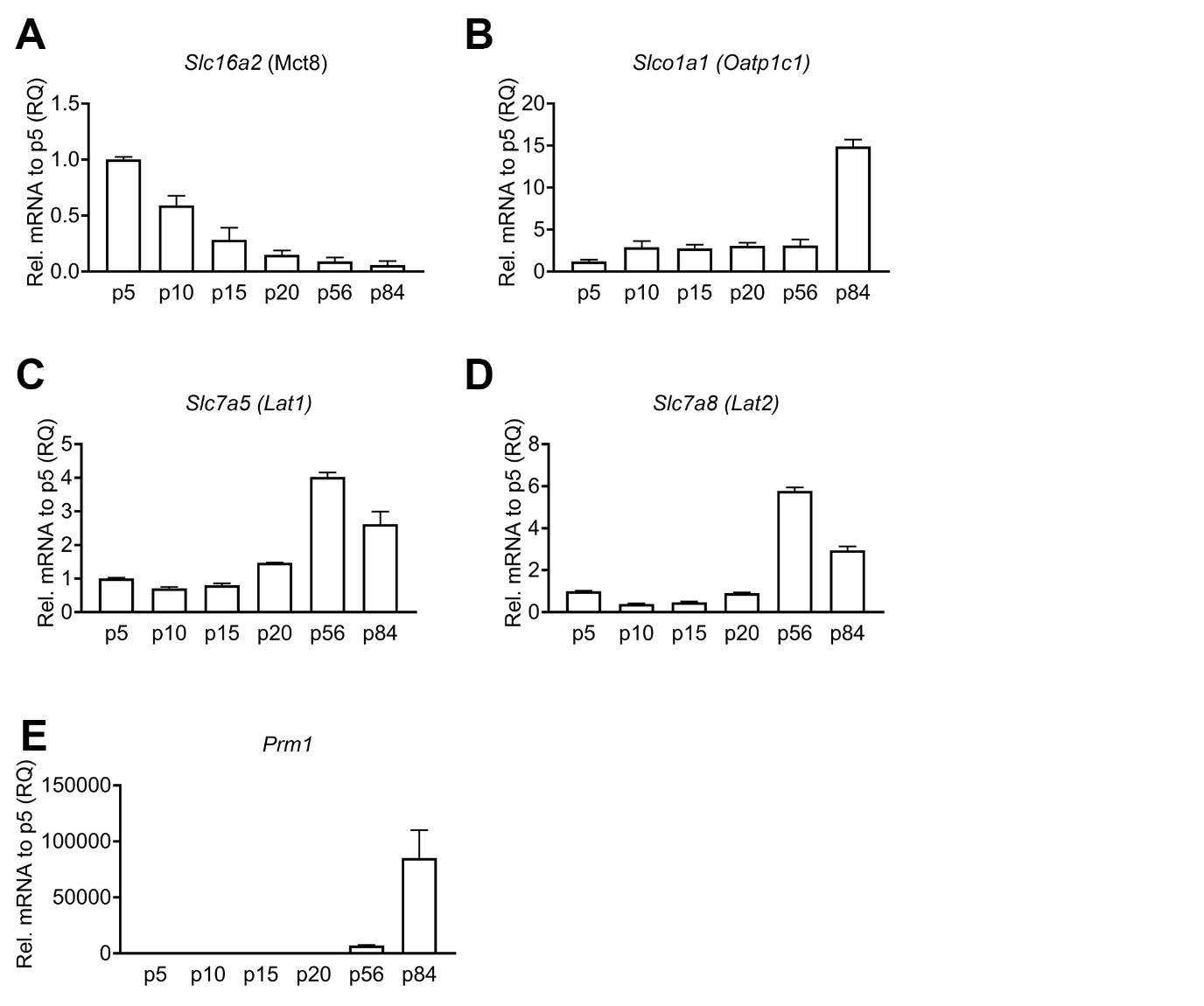
**

**Supplementary Figure 3. Gene expression analysis from *Slc16a2*+/+ male rat testis during development (related to Figure 1)**

(A-D) qRT-PCR analysis of thyroid hormone transporter genes, (A) *Slc16a2*, (B) *Slco1a1*, (C) *Slc7a5*, (D) *Slc7a8* and late stage testis specific gene, Prm1 from postnatal day 5 to day 84. *n*=3

**Supplementary Figure 4. Fertility analysis from *Slc16a2*+/+ and *Slc16a2*-/- male rats**

Litter size of pups produced by *Slc16a2*+/+ females (F) mated with either *Slc16a2*+/+ (*n*=7) or *Slc16a2*-/- (*n*=5) males (M).


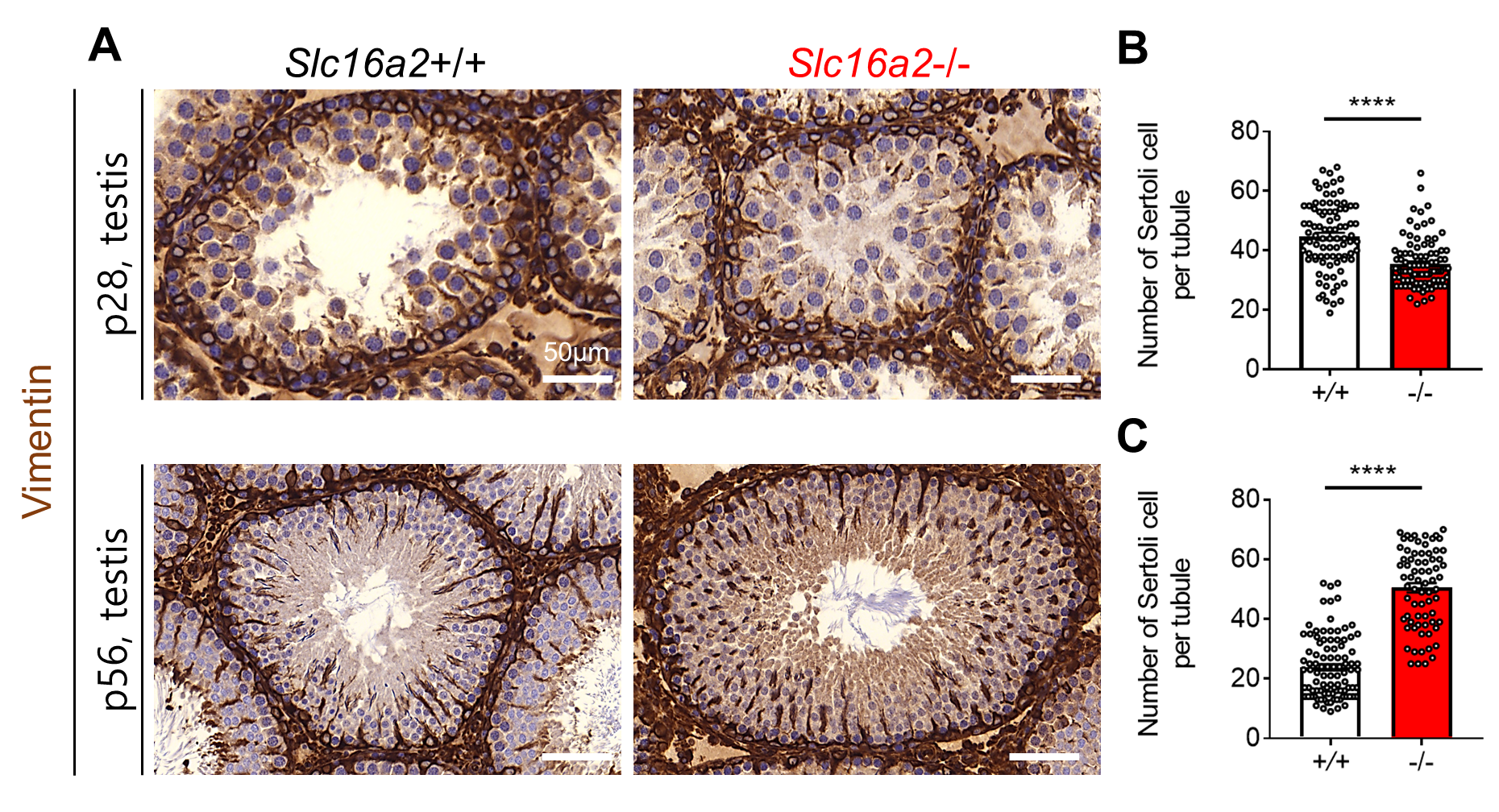


**Supplementary Figure 5. Sertoli cell numbers of *Slc16a2*+/+ and *Slc16a2*-/- male rats**

(A) Representative images of testis sections from postnatal day 28 and 56 of *Slc16a2*+/+ and *Slc16a2*-/- rats that are stained against Vimentin. (B-C) Number of Vimentin-positive cells (Sertoli cells) per seminiferous tubules of *Slc16a2*+/+ and *Slc16a2*-/- at (B) p28 and (C) p56. *n*=4. *P*<0.0001;****


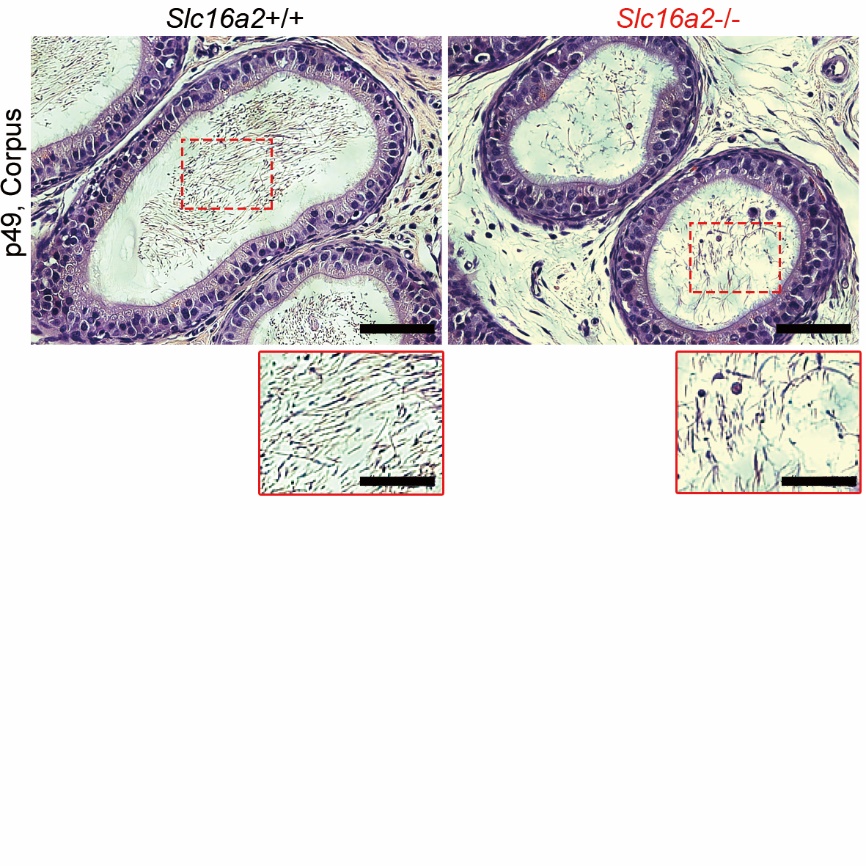


**Supplementary Figure 6. Abnormal sperm morphology and reduced sperm number in *Slc16a2*-/- corpus epididymis**

Representative images of H&E stained corpus epididymis sections of Slc16a2+/+ and *Slc16a2*-/- rats at p49.


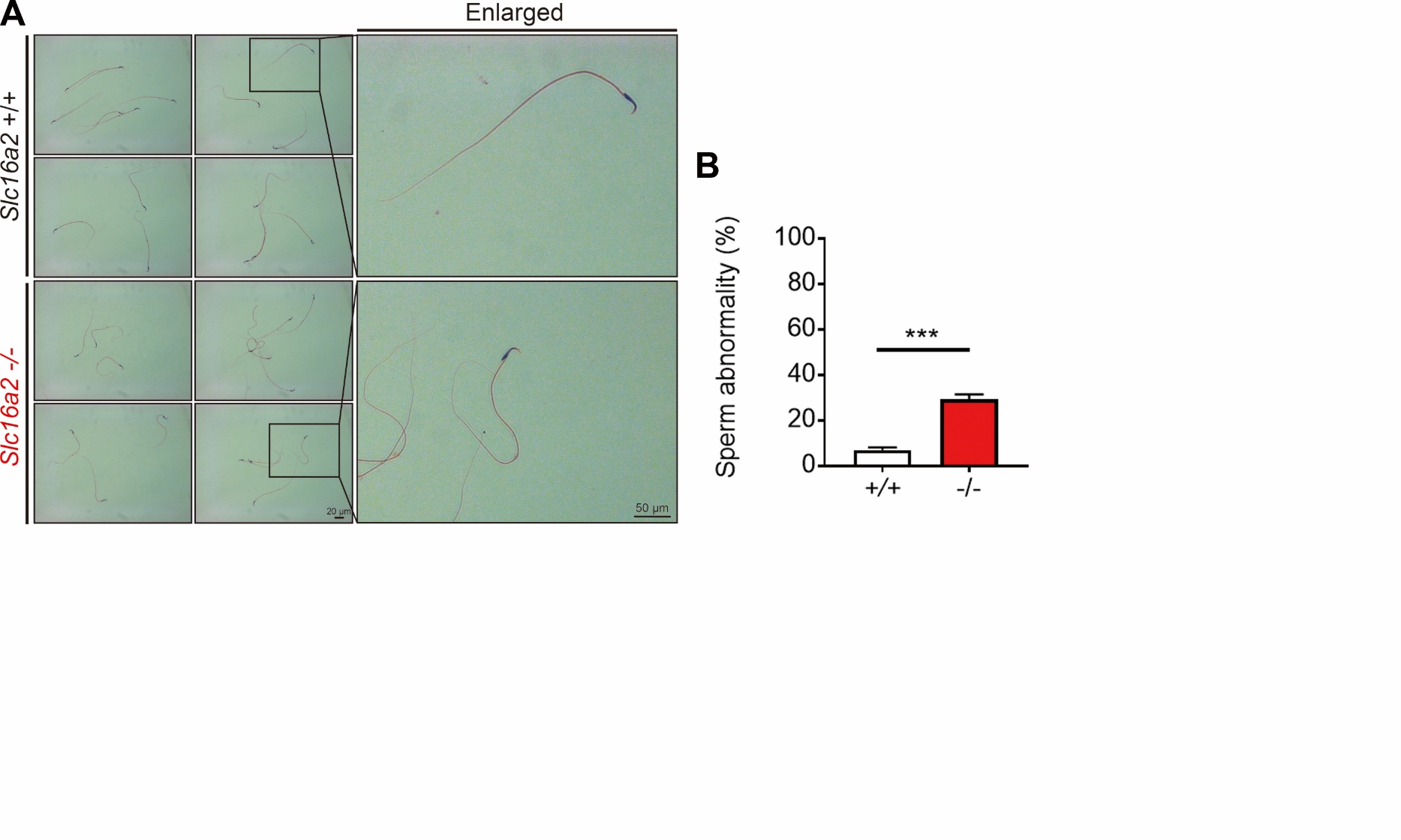


**Supplementary Figure 7. Abnormal sperm structure from *Slc16a2*-/- male rats**

(A) Caudal epididymal spermatozoa of *Slc16a2*+/+ and *Slc16a2*-/- rats. Note that sperms from *Slc16a2*-/- rats showing abnormal tail (sperm tails bent forward in the midpiece). (B) The proportions of abnormal sperm of *Slc16a2*+/+ and *Slc16a2*-/- rats. *n*=3. *P*<0.001;***

**Supplementary Movies**

Supplementary Movie 1. Motility of *Slc16a2*+/+ spermatozoa

Supplementary Movie 2. Motility of *Slc16a2*-/- spermatozoa
